# Breast Implant Capsule: A Murine Model Comparing Capsular Contracture Susceptibility Among Six Breast Implants Available in the Market

**DOI:** 10.1007/s00266-023-03323-0

**Published:** 2023-04-06

**Authors:** Carlos Bérniz, Francisco Carmona-Torre, Cristina Gómez-Martínez, Leire Fernéndez-Ciriza, Jose Luis del Pozo, Bernardo Hontanilla

**Affiliations:** 1 Department of Plastic and Reconstructive Surgery, Clinica Universidad de Navarra, Av. Pio XII 36, 31008 Pamplona, Spain; 2 Infectious Diseases Service, Clínica Universidad de Navarra, Pamplona, Spain; 3 Microbiology Department, Clínica Universidad de Navarra, Pamplona, Spain; 4 IdiSNA, Navarra Institute for Health Research, Pamplona, Spain

## Abstract

**Background:**

Breast implant capsule development and behavior are mainly determined by implant surface combined with other external factors such as intraoperative contamination, radiation or concomitant pharmacologic treatment. Thus, there are several diseases: capsular contracture, breast implant illness or Breast Implant-Associated Anaplastic Large Cell Lymphoma (BIA-ALCL), that have been correlated with the specific type of implant placed. This is the first study to compare all major implant and texture models available in the market on the development and behave of the capsules. Through a histopathological analysis, we compared the behavior of different implant surfaces and how different cellular and histological properties give rise to different susceptibilities to develop capsular contracture among these devices.

**Methods:**

A total of 48 Wistar female rats were used to implant 6 different types of breast implants. Mentor®, McGhan®, Polytech polyurethane®, Xtralane®, Motiva® and Natrelle Smooth® implants were employed; 20 rats received Motiva®, Xtralane® and Polytech polyurethane®, and 28 rats received Mentor®, McGhan® and Natrelle Smooth® implants. The capsules were removed five weeks after the implants placement. Further histological analysis compared capsule composition, collagen density and cellularity.

**Results:**

High texturization implants showed the highest levels of collagen and cellularity along the capsule. However, polyurethane implants capsules behaved differently regarding capsule composition, with the thickest capsules but fewer collagen and myofibroblasts than expected, despite being generally considered as a macrotexturized implant. Nanotextured implants and microtextured implants histological findings showed similar characteristics and less susceptibility to develop a capsular contracture compared with smooth implants.

**Conclusions:**

This study shows the relevance of the breast implant surface on the definitive capsules’ development, since this is one of the most differentiated factors that determine the incidence of capsular contracture and probably other diseases like BIA-ALCL. A correlation of these findings with clinical cases will help to unify implant classification criteria based on their shell and their estimated incidence of capsule-associated pathologies. Up to this point, the establishment of additional groups is recommended as nanotexturized implants seem to behave differently to pure smooth surfaces and polyurethane implants present diverse features from macro- or microtextured implants.

**No Level Assigned:**

This journal requires that authors assign a level of evidence to each submission to which Evidence-Based Medicine rankings are applicable. This excludes Review Articles, Book Reviews, and manuscripts that concern Basic Science, Animal Studies, Cadaver Studies, and Experimental Studies. For a full description of these Evidence-Based Medicine ratings, please refer to the Table of Contents or the online Instructions to Authors www.springer.com/00266.

## Introduction

Since the first generation of breast implants manufactured by the Dow Corning Corporation in 1964, six generations of implants have been developed one after another with benefits and drawbacks associated.

The first generation of implants, with a smooth outer shell made of a silicone elastomer of 0.75 mm thickness, had unacceptable rates of capsular contracture and rupture of the implant [1]. The second generation of implants with a thinner shell came with silicone bleeding and migration to the lymphatic nodes [2]. In 1976, this high complication rate and prevalence of complications associated with breast implants made the Food and Drug Administration (FDA) to impose some new standards helping the development of what would be next generations of the prostheses with fewer expected comorbidities [3].

It took almost 20 years to include a texturization in fourth-generation implants with two different methods: “salt-loss” (e.g., Allergan Biocell®, but also Eurosilicone® or Silimed®) and “imprint stamping” (e.g., Mentor Siltex® surface) that have carried an impact to our days, related to the differences among the two methods.

Until the present day, this road is plagued with dark episodes and low points that include moments when regulations and quality standards fell too short (PIP prostheses [4]) and others when measures were too severe, as time has later shown (FDA’s moratorium on silicone implants [5, 6]).

Almost six decades have passed since the first breast implant was developed and we can only see how still some dogmas are put on doubt. Three big concerns affect both patients and physicians including capsular contracture, BIALC-L and Breast Implant Illness (BII) or more specifically ASIA syndrome (autoimmune/inflammatory syndrome induced by adjuvants).

The objective of this study was to recreate a sample representing the current market of breast implants using a rat model that allowed the controlled development of implant capsules free from any bacterial contamination or other external factors. Histological characteristics have been thoroughly described regarding pathologic and physiologic capsules in breast implants [7]. Previous studies have demonstrated the correlation between capsule thickness and Baker grade, finding that Baker III and IV capsules were significantly thicker than Baker I and II capsules [8, 9]. Regarding capsule contractility, this is mediated by the myofibroblasts attached to the surrounding extracellular tissue. For this reason, the number of myofibroblasts correlates with the incidence and severity of capsular contracture [9, 10]. Regarding inflammation and cellular composition of the capsule, the number of macrophages and monocytes also plays a role in the development of fibrosis in the capsule tissue [11, 12]. Through a histopathological analysis, we compared the behavior of different implant surfaces and how this makes them more or less susceptible to the posterior development of capsular contracture, regardless of other frequently associated factors such as bacterial contamination, particle shedding or radiation.

## Material and Methods

### Animals and Laboratory Conditions

For this study, we used 48 Wistar female rats of 250 g. The experimental protocol was approved by the Ethics Committee of University of Navarra (proyect number 112/14). The animals were housed in groups of 3 to 4 animals, with food, water and controlled temperature and day/night cycle. Every aspect of animal housing and experimentation was considered to minimize suffering, and treatment was according to the European Commission.

This work was supported by Plan Estatal I+D+I from the Instituto Español de Salud Carlos III–Subdirección General de Evaluación y Fomento de la investigación–FEDER (Grant number PI17/00974).

### Study Design

We obtained from different implants surface disks of McGhan texturized BIOCELL ® (macrotexturization), Mentor Siltex ® (microtexturization), Motiva Silksurface ® (nanotexturization), Xtralane microtextured ® (microtexturization) Polytech Microthane ® (polyurethane macrotexturization) and Natrelle Smooth surface ® (smooth surface). For each implant, two disks of 1 cm diameter were used to represent the surface of the implant on both sides. The two disks were glued using cyanoacrylate adhesive at the center of the disk to minimize any interference with the capsule development. Regarding implant classification in different textures, we considered the two most important factors: roughness and surface area and the 2018 ISO classification [13, 14]**.** According to these, Natrelle Smooth® and Motiva Silksurface® implants may be considered smooth implants; McGhan BIOCELL® macrotextured implants; and Xtralane® and Mentor Siltex® microtextured implants. However, the ANSM 2018 classification considers Mentor Siltex® as macrotextured. Finally, Polytech Microthane® implants with polyurethane surface are classified as macrotextured implants both according surface area and roughness.

Due to lack of space on the rats’ dorsum, each animal received 3 implants in independent subcutaneous pockets. Thus, one group of 28 rats received McGhan®, Mentor® and Natrelle Smooth® surfaces and another group of 20 rats underwent Motiva®, Xtralane® and Polytech® implants placement. The implants remained in place for five weeks before extraction, giving enough time for the development of a physiologic capsule. During this time, daily control of the animal and the wound was accomplished for detection of any sign of infection or wound dehiscence.

### Surgical Procedure

For the procedure, all animals were anesthetize with inhaled isoflurane at a concentration of 2%. This way intraperitoneal injection was avoided, with minimal risk of death and less suffering. After shaving and cleaning the dorsum of the back, three incisions of 1 cm long were made distant enough from each other to avoid communicating each pocket. Once the implant was inserted in the subcutaneous space, the wound was closed with staples making sure that the incision was not over the prosthesis.

Five weeks later after killing the animal, the implants with the capsule were removed under sterile conditions. The implants were kept in saline and brought straight to the laboratory for culture and histopathological analysis.

### Microbiological Analysis

Quantitative cultures of the implants and capsules were performed on Columbia agar + 5% sheep blood after vortexing for 30 seconds, sonication for 5 minutes and other 30 seconds of vortexing in 1 ml of BHI to rule out any contamination of the implant. Prolonged cultures in 1 ml of BHI were performed for 10 days to confirm the absence of microbiological isolates on the implants.

### Macroscopic Analysis

After five weeks, the animal was killed and the implant withdrawn. The capsule was separated from the implant, and subsequently, the capsule’s thickness was obtained by the average of four random cross-sectional thickness (ZEISS Axiolab 5 microscope). These measurements were taken by a blind experimenter and were used as a correlation of clinical experience during breast implant explantation as the aspect and thickness may identify an altered or contracted capsule.

### Histological Analysis

The capsules were processed by the Pathology Department of University Clinic of Navarra. Hematoxylin–Eosin staining was used for comparing cellularity and inflammatory cells. For the collagen percentage and composition of the capsule, two methods were used. Sirius red was used to analyze the general amount of collagen present in each capsule, and for a more specific analysis, distinguishing between type 1 and 3 collagen antibodies staining were used (anti-collagen I antibody (ab254113) Abcam INC; anti-collagen **III** antibody [1E7-D7/Col3] (ab23445) Abcam INC). For collagen I, Autostainerlink 48 (Dako) system was used in a 1/100 dilution with an incubation time of 20’. For collagen III, BenchMark Ultra (Roche) system was used in a 1/100 dilution with an incubation time of 16´.

To assess the number of myofibroblasts present, the samples were stained with alfa-SMA (Actin, Muscle Specific (HHF35) Mouse Monoclonal Antibody Cell MarqueTM) and to count for monocytes and macrophages; specifically, CD68 (Anti-CD68 antibody ab125212, ABCAM INC) antibody staining was used. For alfa-SMA, the prediluted antibody was used in a BenchMark Ultra (Roche) system with an incubation time of 1h 8´. For the CD68, an Autostainerlink 48 (Dako) system was used in a 1/100 dilution with an incubation time of 20´. Once each sample was prepared, these were scanned and a software developed at the CIMA (Applied Medical Investigation Center) of University of Navarra was used with the FIJI software [15].

For a blind assessment, the segment of the scanned histologic image of each capsule was selected, and then, the analysis with the software was performed by another investigator who did not know the implant where the capsule was coming from.

### Statistical Analysis

The statistical analysis of the results was performed with SPSS software. The sample of data was first tested to assess normality and then to compare each parameter between different types of implant. Either ANOVA (analysis of variance) and Kruskal–Wallis test was performed for independent samples. The statistical significance was set at *p* < 0.05. For intragroup comparison between each type of implant a Tamhane test was performed setting the statistical significance at *p* < 0.05.

## Results

Mean values for each parameter and each implant are summarized in Table 1.

**Table 1:** Mean values for each parameter studied. Values are arranged in an increasing fashion

	Mean values					
Thickness (µ)	Motiva (830.40)	Smooth (899.55)	Xtralane (1082.50)	Mentor (1295.50)	McGhan (1443.31)	PU (1586.35)
Col I (%/mm^2^)	Smooth (67.52)	McGhan (74.78)	Mentor (75.99)	PU (80.69)	Motiva (88.93)	Xtralane (91.10)
Col III (%/mm^2^)	PU (17.04)	Smooth (18.54)	Xtralane (19.80)	Motiva (20.15)	Mentor (21.35)	McGhan (26.24)
Myofibroblasts (number/mm^2^)	PU (88.90)	Motiva (104.30)	Xtralane (112.10)	Smooth (120.50)	Mentor (125.16)	McGhan (139.50)
Red Sirius (%/mm^2^)	Smooth (52.16)	Mentor (62.54)	Motiva (63.14)	McGhan (63.19)	PU (63.37)	Xtralane (65.97)
CD-68 (monocytes macrophages/mm^2^)	Smooth (32.82)	PU (41.50)	Motiva (68.10)	Xtralane (91.65)	Mentor (92.89)	McGhan (202.36)
H-E Cellularity (Density grade I – V)	Smooth (1.25)	PU (1.40)	Motiva (2.50)	Xtralane (3.55)	Mentor (4.32)	McGhan (4.86)

### Capsule Thickness

None of the capsules revealed any bacterial growth after 10 days. For capsule thickness assessment, both macroscopic and microscopic measurements were used. When compared, both results were very similar, so for more precision and to avoid redundancy, microscopic data were used. Among all types of implants, significant differences were found (*p *< 0.001) in both macroscopic and microscopic capsule thicknesses (Table 2).

**Table 2:** Statistical differences after Kruskal–Wallis inter-group analysis in all comparisons except for collagen III

	Thickness	Micros	Myofibroblasts	Red Sirius	Collagen I	Collagen III	Monocytes & Macrophages	H–E Cellularity
Chi-Square	43.410	53.773	12.489	14.273	36.315	3.384	27.741	
Statistical significance	0.000*	0.000*	0.029*	0.014*	0.000*	0.641	0.000*	0.000
Degrees of freedom	5	5	5	5	5	5	5	5

*Statistically significant differences

**Fig. 1:**
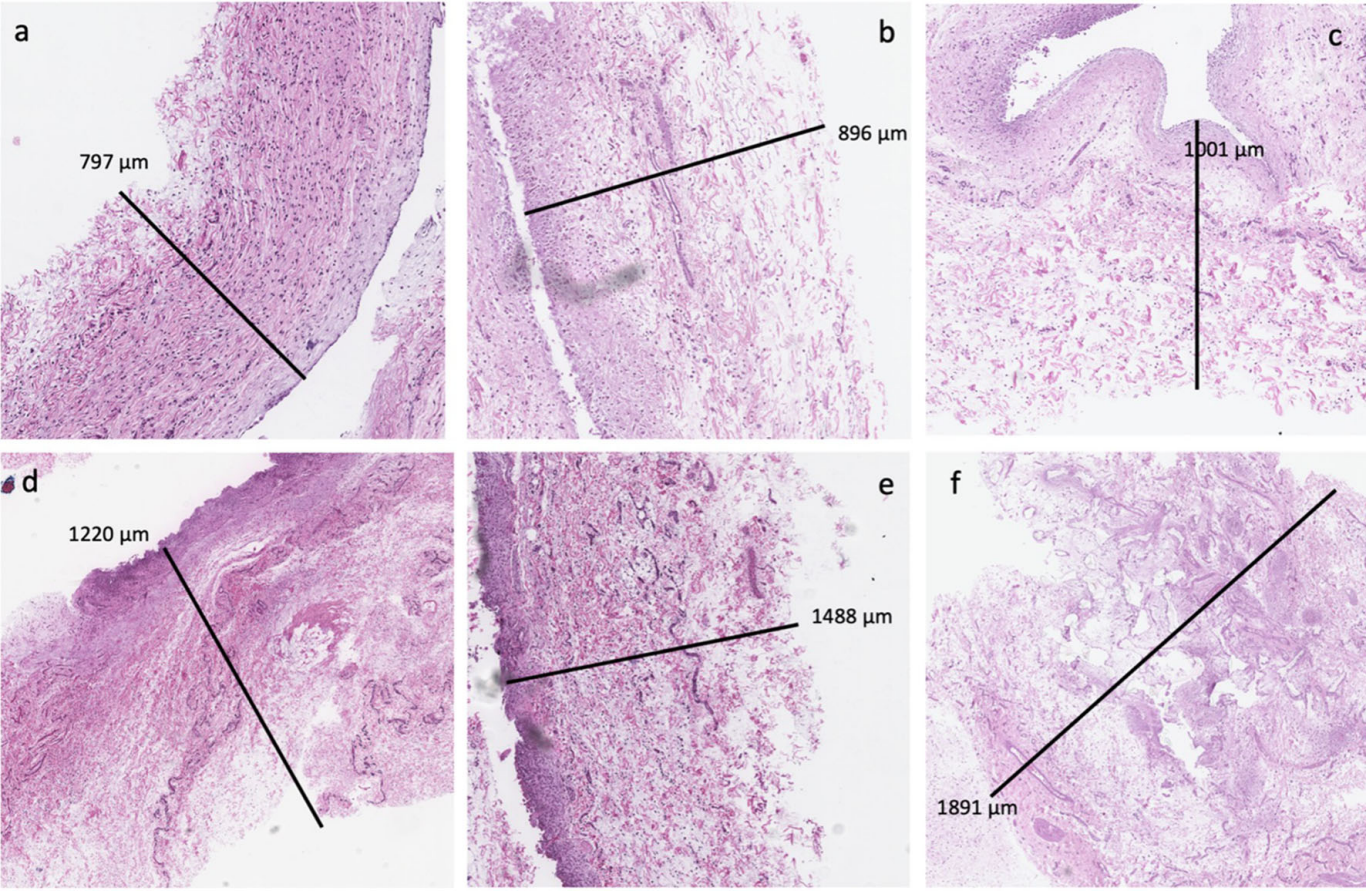
Capsule thickness measured in Hematoxilin–Eosin staining. From thinnest to thickest: Natrelle Smooth (**a**), Motiva (**b**), Xtralane (**c**), Mentor (**d**), McGhan (**e**) and polyurethane (**f**)

The results showed increasing thickness capsule with increasing texturization of implants (Fig. 1). Motiva and smooth implants showed the thinnest capsule and McGhan and polyurethane the thickest. Statistical significance was found after subgroup analysis between the smoothest texturizations (smooth, motiva) and the thickest (McGhan, Mentor, polyurethane). Xtralane implants fell between these two groups closer to the smoothest implants showing only statistical differences with polyurethane and McGhan implants but no with Mentor (Tables 1 and 3). In the macroscopic measurements, there were no significant differences between Xtralane and the other implants.

**Table 3:** Thickness capsule intra-group comparison with all statistically significant results. Bidirectional redundant comparisons have been removed

Implant (I)	Implant (J)	Mean difference (I–J)	Standard error	Significance	Confidence interval (Lower limit)
Xtralane	McGhan	− 378.286	131.9174	0.090	− 785.708
	Polyurethane	− 503.850*	125.0182	0.004	− 894.331
Smooth	McGhan	− 645.689*	117.3381	0.000	− 1007.758
	Mentor	− 474.475*	96.4080	0.000	− 769.942
	Polyurethane	− 771.254*	109.5243	0.000	− 1113.942
McGhan	Motiva	630.386*	118.2662	0.000	264.362
Mentor	Motiva	459.171*	97.5354	0.000	157.879
Motiva	Polyurethane	− 755.950*	110.5180	0.000	− 1103.045

*Statistically significant differences

### Collagen Composition

To study the collagen density of each capsule, three different measures were employed. First red Sirius was used to quantify the total amount of collagen in the capsule. Significant differences were found (*p *< 0.014, Table 1) with smooth implants showing the lowest percentage and similar values for the rest of implants. This was supported with intragroup analysis founding significant differences only between smooth implants and Xtralane, polyurethane and Mentor (Tables 1 and 4).

**Table 4:** Red Sirius intra-group comparison with all statistically significant results. Bidirectional redundant comparisons have been removed

Implant (I)	Implant (J)	Mean difference (I–J)	Standard error	Significance	Confidence interval (Lower limit)
Xtralane	Smooth	13.9034*	4.02953	0.019	1.4195
Smooth	Mentor	− 11.1054	3.69478	0.059	− 22.4283
	Polyurethane	11.3059	3.87344	0.079	− 0.6732

*Statistically significant differences

**Fig. 2:**
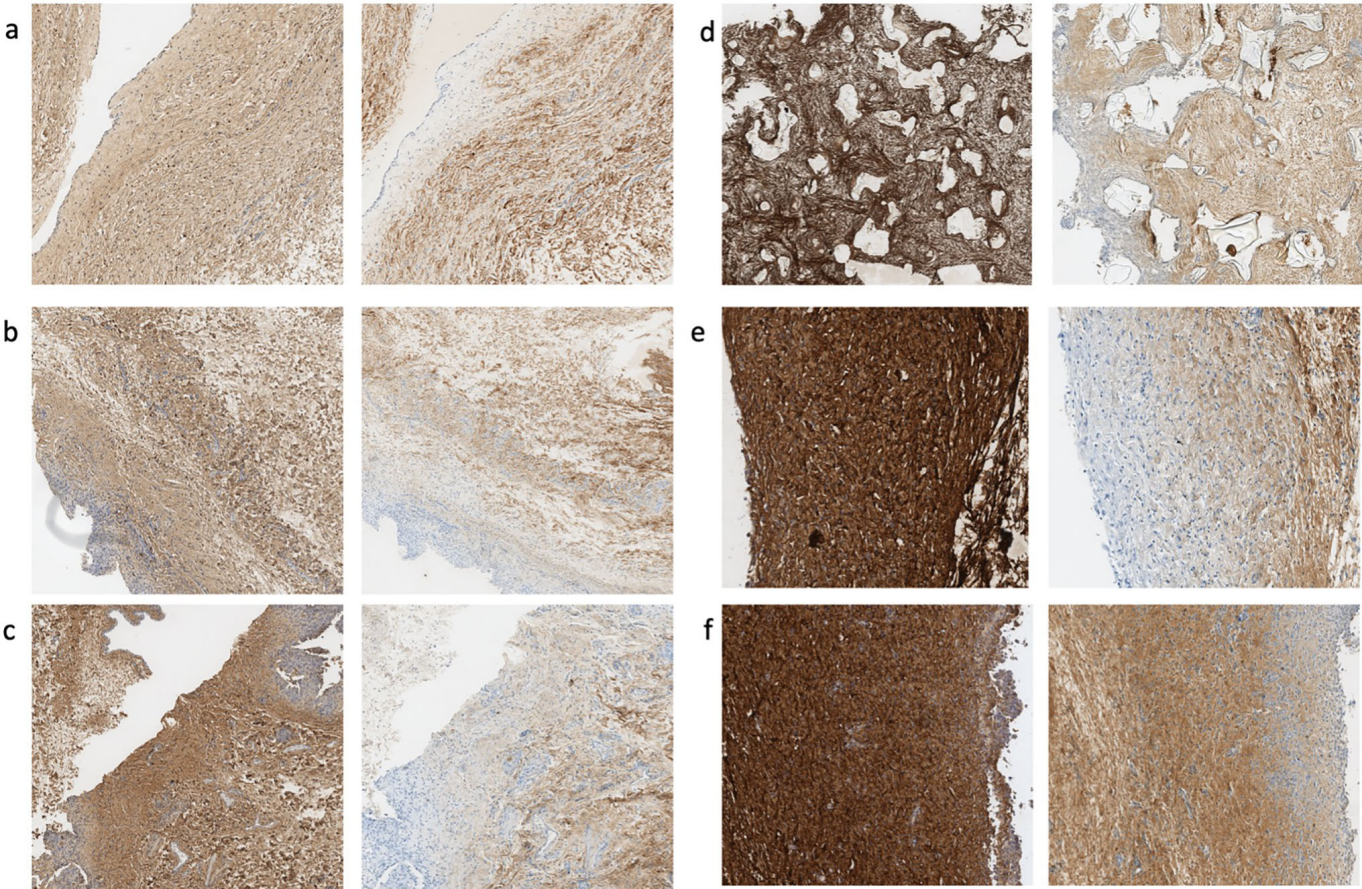
Collagen I (left image) and III (right image) density with antibody staining. Nanotextured implants (**e**, **f**) showed the highest density in collagen I. Natrelle Smooth (**a**), Mentor (**b**), McGhan (**c**), polyurethane (**d**), Motiva (**e**) and Xtralane (**f**)

For a more detailed analysis, collagen I and III antibodies were used (Fig. 2). Collagen I showed significant differences and a greater percentage versus collagen III (Table 2), which also didn’t show any statistical significance between different implants. Collagen I showed its minimal percentage in smooth implants and its higher amount among the low texturization implants (motiva and Xtralane) (Tables 1 and 5). When intragroup analysis was performed, statistical significance was found between these two and the rest of implants but no between the other four types.

**Table 5:** Collagen 1 intra-group comparison with all statistically significant results. Bidirectional redundant comparisons have been removed

Implant (I)	Implant (J)	Mean difference (I–J)	Standard error	Significance	Confidence interval (Lower limit)
Xtralane	McGhan	14.0111*	3.79669	0.011	2.0736
	Smooth	22.3115*	4.16214	0.000	9.1867
	Mentor	11.7265*	3.24756	0.013	1.5726
	Polyurethane	10.4095*	2.02974	0.000	4.0662
Smooth	Motiva	− 20.1460*	4.17870	0.000	− 33.3103
McGhan	Motiva	− 11.8456	3.81484	0.055	− 23.8277
Mentor	Motiva	− 9.5610	3.26876	0.083	− 19.7699
Motiva	Polyurethane	8.2440*	2.06349	0.004	1.7975

*Statistically significant differences

**Fig. 3:**
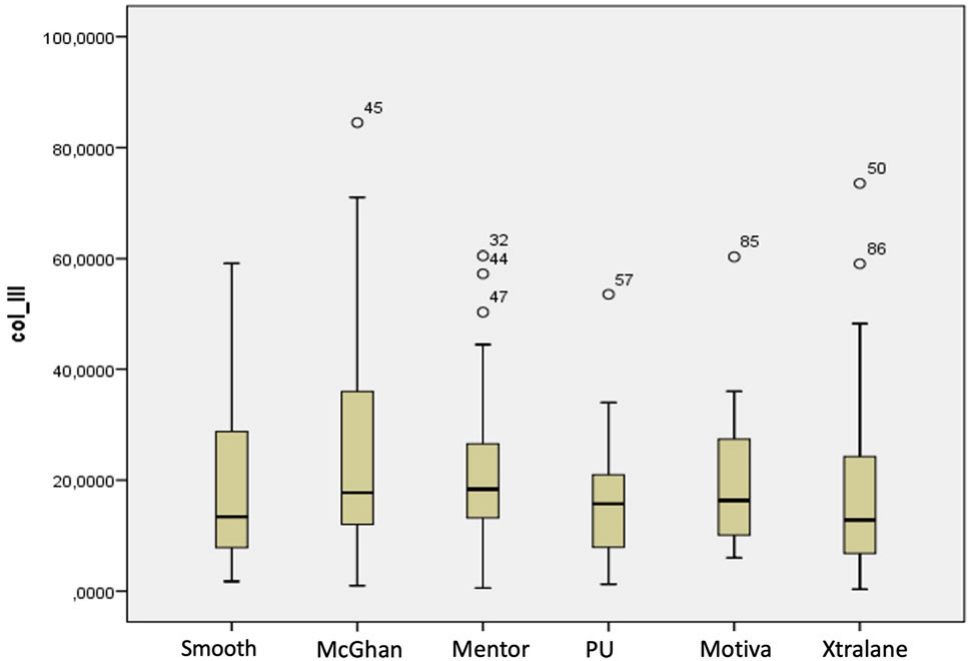
Diagram showing statistical values in collagen III concentration with smooth and polyurethane showing lowest mean values

As mentioned before, collagen III showed an increasing trend from low texturization implants to higher texturization with the exception of polyurethane implants which showed the lowest value. However, these differences were not statistically significant (*p *= 0.641) (Fig. 3).

The disposition of collagen fibers was also assessed, distinguishing between parallel fiber disposition, observed in smooth surface implant and a more anarchic pattern present in the rest of capsules (micro- and macrotextured devices).

**Fig. 4:**
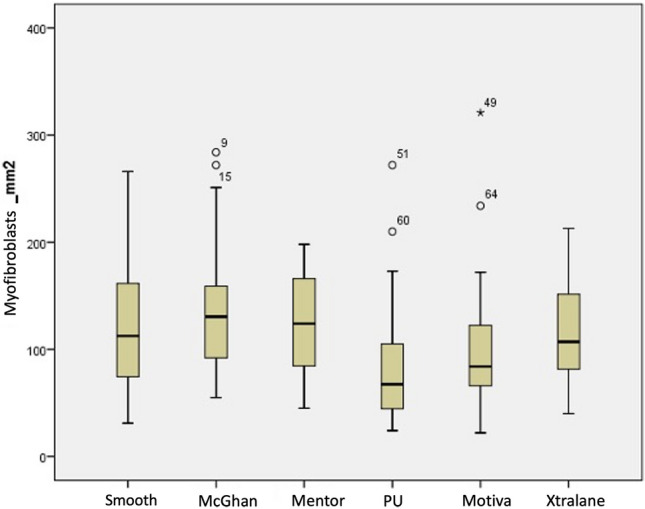
Diagram showing mean values of myofibroblasts numbers with polyurethane prostheses showing the lowest concentration

Finally, number of myofibroblasts per mm^2^ was studied using alfa-SMA staining showing statistical differences (Table 2) and a similar pattern as collagen III with increasing number from low to high texturization but still having the lowest number in the polyurethane implants (Fig. 4, Table 1).

### Cellularity

**Fig. 5:**
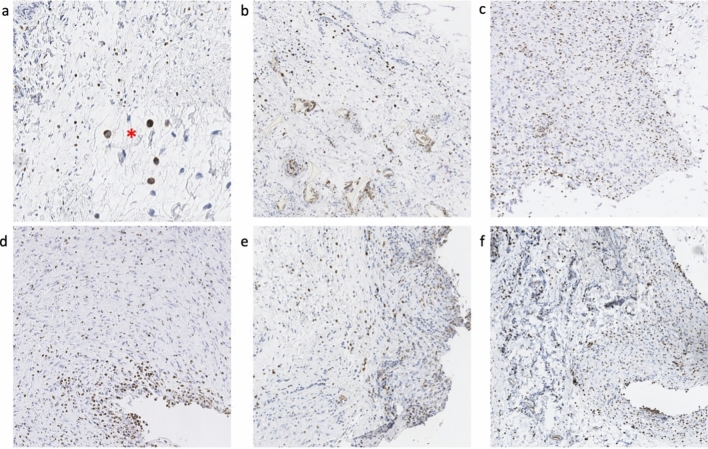
Monocytes and macrophages density with CD68 staining. Lowest density appears in Natrelle Smooth (**a**) and polyurethane implants (**b**). McGhan (**f**) capsule showed the highest value for cellularity significantly higher than Mentor prostheses (**e**). Natrelle Smooth (**a**), polyurethane (**b**), Motiva (**c**), Xtralane (**d**), Mentor (**e**) and McGhan (**f**). Red asterisk shows augmented view of a macrophage (left) and a monocyte (right) in the collagen fibers of the capsule

CD-68 staining for monocytes and macrophages showed significant differences (*p *< 0.001) among the groups with the highest values for Mentor and McGhan implants. However, McGhan capsules showed a number of cells significantly higher than Mentor prosthesis, which presented similar average values to Xtralane implants (Fig. 5). In the subgroup analysis, significant differences were found between smooth implants and the rest except for polyurethane implants that showed slightly higher values than the smooth ones with no significant differences (Tables 1 and 6).

**Table 6:** CD68 intra-group comparison with all statistically significant results. Bidirectional redundant comparisons have been removed

Implant (I)	Implant (J)	Mean difference (I–J)	Standard error	Significance	Confidence interval (Lower limit)
Smooth	Mentor	14.0111*	3.79669	0.004	2.0736
	Motiva	22.3115*	4.16214	0.009	9.1867
	Xtralane	11.7265*	3.24756	0.000	1.5726
	McGhan	10.4095*	2.02974	0.000	4.0662
Mentor	McGhan	− 20.1460*	4.17870	0.079	− 33.3103
Polyurethane	Xtralane	− 11.8456	3.81484	0.015	− 23.8277
	McGhan	− 9.5610	3.26876	0.002	− 19.7699

*Statistically significant differences

Hematoxilin–Eosin staining showed the same distribution and trend for density of inflammatory cells infiltrate with significant differences among all the implants, except for polyurethane implants, which showed similar values as smooth implants with no statistically significant differences between them (Tables 1 and 7).

**Table 7:** H-E intra-group comparison with all statistically significant results. Only smooth and polyurethane implants showed no statistically significant differences. Bidirectional redundant comparisons have been removed

Implant (I)	Implant (J)	Mean Difference (I–J)	Standard Error	Significance	Confidence Interval (Lower limit)	Confidence Interval (Higher limit)
Smooth	McGhan	− 3.607*	0.132	0.000	− 4.05	− 3.16
	Mentor	− 3.071*	0.132	0.000	− 3.52	− 2.63
	Motiva	− 1.250*	0.144	0.000	− 1.74	− 0.76
	Xtralane	− 2.300*	0.144	0.000	− 2.79	− 1.81
McGhan	MENTOR	0.536*	0.132	0.007	0.09	0.98
	Polyurethane	3.457*	0.144	0.000	2.97	3.94
	Motiva	2.357*	0.144	0.000	1.87	2.84
	Xtralane	1.307*	0.144	0.000	0.82	1.79
Mentor	Polyurethane	2.921*	0.144	0.000	2.43	3.41
	Motiva	1.821*	0.144	0.000	1.33	2.31
	Xtralane	0.771*	0.144	0.000	0.28	1.26
Polyurethane	Motiva	− 1.100*	0.156	0.000	− 1.63	− 0.57
	Xtralane	− 2.150*	0.156	0.000	− 2.68	− 1.62
Motiva	Xtralane	− 1.050*	0.156	0.000	− 1.58	-0.52

*Statistically significant differences

## Discussion

In recent years, many concerns have emerged related to breast implant complications. The most frequent, capsular contracture, has shown a clear link with infection, but with great differences depending on type of implant, most specifically the surface of the implant [16, 17]. Breast implant illness (BII) has also become more relevant in the recent years, despite being a more unspecific pathology [18]. Finally, Breast Implant-Associated Large Cell Lymphoma has turned into a great concern for both patients and clinicians with a clear relationship with very specific implant texturizations [19].

While recent literature has tried to clarify the causes and reasons for these pathologies, most of the studies are limited to a small sample of what the current market of breast implants offers. Many authors have compared different implants, contaminated with different microorganisms and sometimes treated with antibiotics to analyze the behavior of the capsule [20]. What all these studies share is the differences in the results depending on the implant surface. Smooth implants have historically been associated with a higher rate of capsular contracture [21]. With the emergence of BIALCL cases and the etiological association with macrotexturization, the use of smooth surface, as well as nanotextured implants, has dramatically increased without an associated increase in capsular contracture and similar rates of patient satisfaction even in reconstructive procedures [22]. Previous literature has backed this, as smooth implants are less prone to gather bacteria or other microorganisms due to its surface [23]. More controversial in this matter are polyurethane implants; some authors have considered them comparable to high textured implants with similar rates of BIALCL, but more thorough revisions show their behavior is completely different and this should be considered out of the “high texturization” group [24, 25].

Capsular contracture has been the most frequent complication, usually requiring reintervention with high morbidity and economic cost for the patient and the physician. The incidence and prevalence of capsular contracture depend on multiple factors [26]: type of implant, placement plane and purpose (aesthetic vs reconstructive procedures [27]). The etiology of capsular contracture remains controversial, but besides direct contamination during inset of the implant, seeding from a distant focus, has also been demonstrated as a possible and preventable cause [20]. As this is clearly a multifactorial issue, we obtained a sample of capsules of six different implants to study their characteristics and susceptibility to develop a capsular contracture depending on its surface regardless of frequent concomitant factors such as radiotherapy or bacterial contamination.

**Fig. 6:**
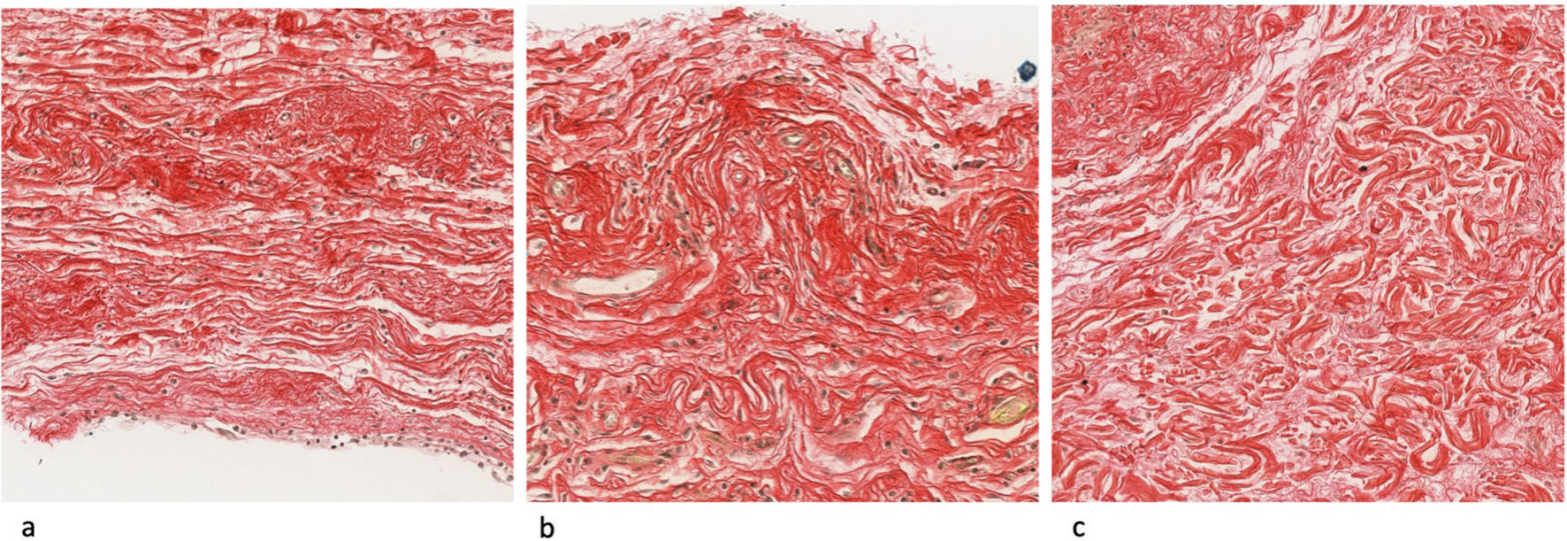
Comparison of collagen fibers disposition in smooth surface capsule (**a**) Natrelle Smooth vs Mentor Siltex (**b**) and McGhan Biocell (**c**). The latter show a much more anarchic disposition compared to the parallel disposition of the fibers in the first image

Smooth implants have shown in multiple previous studies to develop thinner and less inflammatory capsules, despite when a complication occurs, or the implant is contaminated these are more susceptible to develop a capsular contracture. For this reason, there has classically been a correlation between smooth implants and capsular contracture [28, 29]. Our findings corroborate this behavior, with Natrelle Smooth surface implants presenting the thinnest capsules, with the lowest percentages of collagen I and III, and lowest values for cellularity in Hematoxilin–Eosin and CD68 staining; however, this last marker (CD68 positive macrophages) has been inversely correlated with the deposit of collagen [12]. As mentioned, smooth implants have gained popularity in the last years due to BIALCL, and while previous studies reported a significant higher incidence of capsular contracture, recent literature reinforces the idea that this difference is only significant when implants are placed over the muscle [21]. This is probably because surgical technique has refined through the years to the finest expression, with “no touch” technique using funnels and other tools being mandatory. Our histopathological analysis supports the higher susceptibility of smooth implants to develop a capsular contracture related to its architecture, composition (rich in myofibroblasts) and collagen fibers parallel disposition. First texturized implants were developed to diminish the incidence of capsular contracture by altering the vectors of the forces around the implant, but also by altering the composition and tissue structure of the capsule. Myofibroblasts adhesion is enhanced in smooth surface implants due to the expression of higher levels of proteins such as vinculin or focal adhesion kinase [30]. In addition, this higher density of myofibroblasts in smooth surface capsules (similar to textured implants in our sample, without statistically significant differences) tends to proliferate in the outer part of the tissue along parallel fibers of collagen as observed in our sample (Fig. 6a). This disposition of the tissue, more disorganized in textured implants, explains why when the myofibroblasts are stimulated in smooth implants create stronger forces leading to capsular contracture when other factors are present (Fig. 6b, c).

**Fig. 7:**
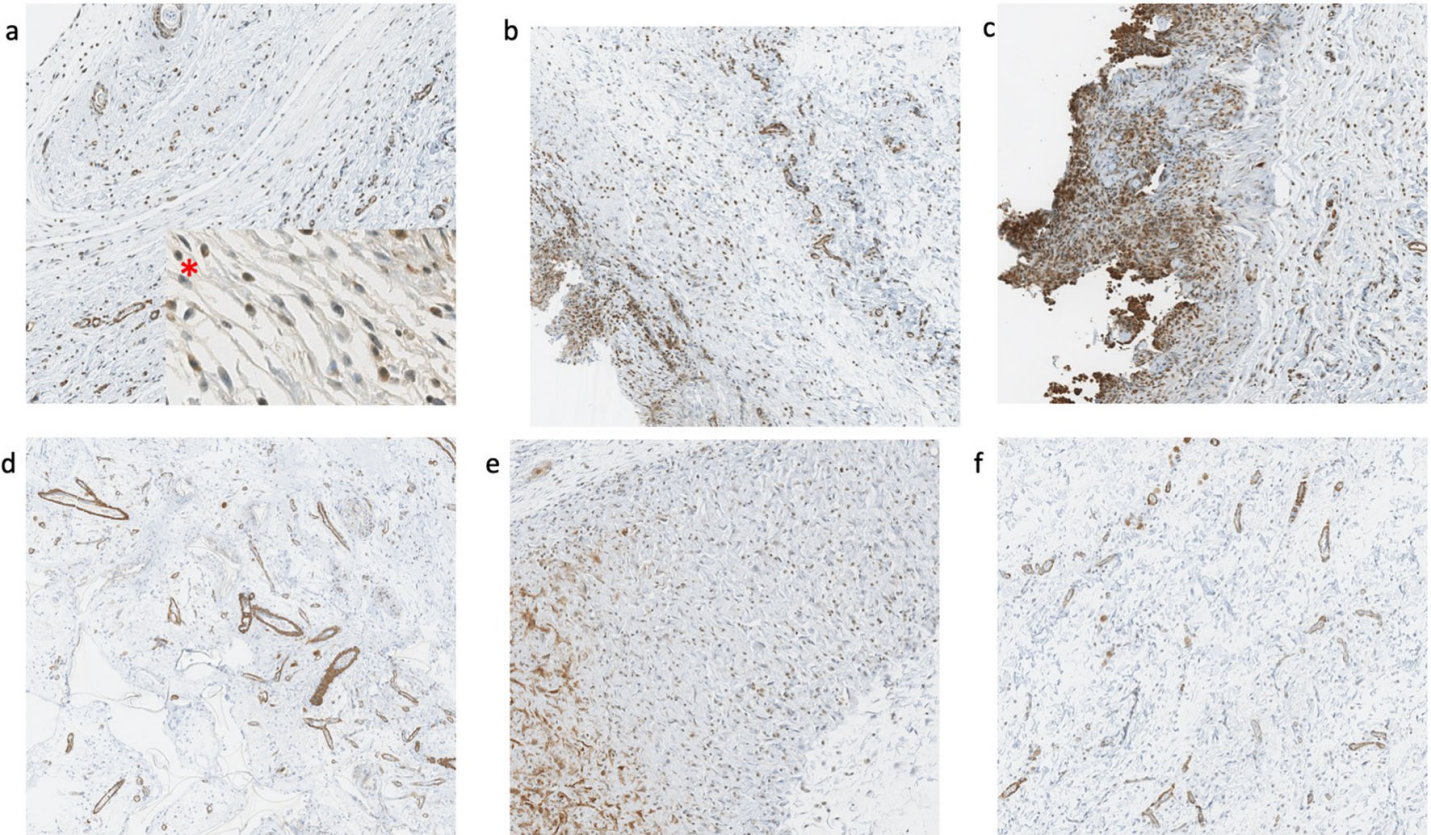
Myofibroblasts density per mm^2^ showing an increasing trend from lowest to highest texturization with the exception of polyurethane implants (**d**). Natrelle Smooth (**a**), Mentor (**b**), McGhan (**c**), polyurethane (**d**), Motiva (**e**) and Xtralane (**f**). Red asterisk shows detailed image of myofibroblasts along the collagen fibers

For the development of a capsule, three elements must be present, a thick capsule, composed of parallel oriented collagen fibers and rich in contractile myofibroblasts [9]. This slower development of the capsule in texturized implants allows a more anarchic pattern in the distribution of collagen fibers, explaining the resistance to capsular contracture despite the high cellularity and capsule thickness [31]. In our sample, texturized implants (i.e., Mentor Siltex and McGhan Biocell) showed these characteristics regarding the collagen composition of the capsule with the highest levels of collagen III. As previous literature show, this may be related to a more immature tissue [32]. Despite presenting the thickest capsules with the higher density of myofibroblasts, monocytes and macrophages (Figs. 1, 2, 5 and 7), the composition and collagen fiber structure of the capsule relate to the lower susceptibility of texturized implants surfaces to develop a capsular contracture. Mentor and McGhan implants only showed significant differences in the number of monocytes and macrophages and total number of inflammatory cells. The salt-loss technique of BIOCELL surface creates a more irregular and coarser surface by pressing a fine salt against the silicone followed by a laminar flow to create an irregular pattern [33]. This is different from the “imprint stamping” of Siltex surface that works by pressing the uncured silicone against a polyurethane foam [34]. The former surface allows for a larger area to host a higher number of cells and has been linked with the development of BIALCL; this implant surface is no longer available in the market, forcing the company to replace this for smooth textured devices.

Nanotextured implants and microtextured implants are presented today by many surgeons as the safest option, maintaining the advantages of textured implants regarding capsular contracture, without the concerns of BIALCL. In our sample, both Motiva Silksurface (in our sample considered nanotextured, despite classified as smooth texture in the ISO classification) and Xtralane behaved similarly regarding thickness (halfway between smooth and texturized implants) and cellularity as well as capsule composition. With our results, we found that Motiva Silksurface and other similar implants should probably fall in the microtextured group in international classifications such as the ISO, as the histological behavior is closer to the Xtralane microtextured implant than the Natrelle Smooth. The only difference between these two surfaces was the Xtralane higher levels of collagen I and total collagen, what may translate into a more mature capsule**.** In other parameters, despite behaving similarly, Xtralane presented a thicker capsule than Motiva implants, richer in myofibroblasts, CD-68 cells and cellularity.

In our study, polyurethane-covered implants showed the thickest capsule, with a high percentage of collagen, with the lowest fraction of collagen type III, even lower than smooth implants. The collagen fibers disposition, as this is integrated between the polyurethane matrix, is completely disorganized and anarchic, explaining the low incidence of capsular contracture of this type of implant [35] . In addition, PU-capsule showed the lowest levels of myofibroblast, diminishing the contractile capacity of the tissue against an inflammatory response. As time passes and the polyurethane is absorbed, a smooth implant is left inside a capsule that is more similar to the one of a texturized implant. That may be the reason why previous long-term reports observed a slight peak in the incidence of capsular contracture with PU-coated implants between years six and fifteen after implantation, despite always lower than smooth implants [36]. Many classifications have considered PU-implants as the highest texturization implants. At this point, it is of paramount importance to point out the multiple and different classifications of what a high texturization implant is [37]. As mentioned before and as a matter of consensus, we relied on the ISO classification [14]. According to it, there would be three types of surfaces depending on the roughness measured in µm: smooth (< 10 µm), microtextured (10–50 µm) and macro-textured (> 50 µm). This only considers the roughness, not the thickness; the peaks and valleys that different methods for texturization create on the surface of the implant. At this respect, as many authors have clearly explained, polyurethane implants shouldn’t be considered as macrotextured surface devices [25]. The outer shell of polyurethane forms a matrix that integrates with the surrounding tissues, sticking to them in a “velcro” effect and slowly dissolving with the years. This matrix is a three-dimensional space, different from any other textured bidimensional surface implants, and for that reason it should be classified apart from micro- or macrotextured.

This study recreates a scenario that can be improved and expanded for the comparison of different prostheses. Rats have an immune system and an inflammatory and cellular response different from humans, so maybe a model with a larger animal could simulate more accurately the development conditions of the implant capsule. The other limitation is the duration of the study; 5 weeks is a short time for a capsule contracture to develop, polyurethane surface to degrade and of course BIALCL to grow, but it is enough for the complete formation of a capsule that is the object for study and comparison, as this would be very difficult to reproduce in a human model, since different implants are inserted in the same specimen. On the other hand, we believe that the exhaustive histopathological analysis and the wide sample of devices studied provided new information and may be developed to be as close as possible to the clinical model in future studies.

## Conclusions

The structure and composition of the capsule among all the implants used in the study are consistent with previous literature related to the development of capsular contracture.

1. Smooth implants (Natrelle Smooth surface®) present more susceptibility to develop a capsular contracture, due to its architecture (parallel oriented collagen fibers) and higher density of myofibroblasts.
2. Macro-texturized implants (McGhan texturized BIOCELL®) generate a stronger cellular response, but its structure and composition are protective against the development of a capsular contracture.
3. Nanotextured (Motiva Silksurface®) and microtextured (Mentor Siltex®, Xtralane Microtextured®) implants offer the advantages of smooth implants with less susceptibility to develop capsular contracture according to their histopathological characteristics (low levels of cellularity and CD-68 cells with lower number of myofibroblasts).
4. We demonstrated clear differences among breast implant capsule composition, and these are related to the morbidity associated with the use of prosthetic devices.
5. Based on our results, we recommend adopting the ISO classification as a standardized system. This is a good measure to unify implant classification criteria depending on the shell, but given the results of our study, the establishment of additional groups is recommended:

(a) Nanotexturized implants shouldn’t fall in the pure smooth group as the observed behavior and histological characteristics are closer to microtexturized implants.(b) Polyurethane implants should fall in a different category from macro- or microtextured implants, as their behavior and the histopathological findings give them their own and specific characteristics.
